# Prevalence of asthma in Portugal - The Portuguese National Asthma Survey

**DOI:** 10.1186/2045-7022-2-15

**Published:** 2012-08-29

**Authors:** Ana Sa-Sousa, Mário Morais-Almeida, Luis Filipe Azevedo, Rosa Carvalho, Tiago Jacinto, Ana Todo-Bom, Carlos Loureiro, António Bugalho-Almeida, Jean Bousquet, João Almeida Fonseca

**Affiliations:** 1 Center for Research in Health Technologies and Information Systems, Faculdade de Medicina da Universidade do Porto, Porto, Portugal; 2 Allergy and Clinical Immunology Division Unit, Hospital CUF-Descobertas, Lisbon, Portugal; 3 Sociedade Portuguesa de Alergologia e Imunologia Clínica, LIsbon, Portugal; 4 Health Information and Decision Sciences Department, Faculdade de Medicina da Universidade do Porto, Porto, Portugal; 5 GBN, Estudos de Mercado, Porto, Portugal; 6 Allergy Unit, Hospital and Institute CUF, Porto, Portugal; 7 Allergy and Clinical Immunology Division, Hospitais da Universidade de Coimbra, Coimbra, Portugal; 8 Clínica Universitária de Pneumologia, Faculdade de Medicina da Universidade de Lisboa, Lisbon, Portugal; 9 Coordinator of Comissão de Acompanhamento do Programa Nacional de Controlo da Asma, Lisbon, Portugal; 10 Hôpital Arnaud de Villeneuve, Centre Hospitalier Universitaire Montpellier, Montpellier, France; 11 Allergy and Clinical Immunology Division, Hospital S. João EPE, Porto, Portugal

**Keywords:** Asthma, Computer-assisted-telephone–interviewing (CATI), Epidemiology, Prevalence

## Abstract

**Background:**

Asthma is a frequent chronic respiratory disease in both children and adults. However, few data on asthma prevalence are available in Portugal. The Portuguese National Asthma Survey is the first nationwide study that uses standardized methods. We aimed to estimate the prevalence of current asthma in the Portuguese population and to assess the association between ‘Current asthma’ and comorbidities such as upper airways disease.

**Methods:**

A cross-sectional, population-based, telephone interview survey including all municipalities of Portugal was undertaken. Participants were randomly selected to answer a questionnaire based on the Portuguese version of the GA^2^LEN survey. ‘Current asthma’ was defined as self-reported lifetime asthma and at least one of 3 symptoms in the last 12 months: wheezing, waking with breathlessness or having an asthma attack.

**Results:**

Data were obtained for 6 003 respondents, with mean age of 38.9 (95%CI 38.2-39.6) years and 57.3% females. In the Portuguese population, the prevalence of ‘Current asthma’ was 6.8% (95%CI 6.0-7.7) and of ‘Lifetime asthma’ was 10.5% (95%CI 9.5-11.6) Using GA^2^LEN definition for asthma, our prevalence estimate was 7.8% (95%CI 7.0-8.8). Rhinitis had a strong association with asthma (Adjusted OR 3.87, 95%CI 2.90-5.18) and the association between upper airway diseases and asthma was stronger in patients with both rhinitis and sinusitis (Adjusted OR 13.93, 95%CI 6.60-29.44).

**Conclusions:**

Current asthma affects 695 000 Portuguese, with a prevalence of 6.8%. People who reported both rhinitis and sinusitis had the highest risk of having asthma.

## Background

Asthma is a chronic inflammatory disease of the airways associated with episodes of wheezing, chest tightness, shortness of breath and cough especially at night [1]. Epidemiological studies at a population level are crucial for the assessment of population needs related to chronic respiratory diseases, the baseline to define health policies [2]. However few data on asthma prevalence are available in Portugal and, as most of the studies use non-standardized methods, prevalence estimates are difficult to compare. Standardized methodology on asthma symptoms prevalence is limited for ages between 20–44 years old in *European Community Respiratory Health Survey* (ECRHS) [3] and 6–7 and 13–14 years old in *International Study of Asthma and Allergies in Childhood* (ISAAC) [4]. In Portugal, both surveys were conducted in a small number of cities [5,6]. Recently, the EU-funded *Global Allergy and Asthma European Network* (GA^2^LEN) is conducting a large survey on the prevalence of airway diseases, built on methods used in earlier studies (mainly ECRHS), however only one Portuguese city was included [7]. The majority of the asthma studies in Portugal were done on school-attending children and teenagers from selected cities or regions [8-15]. For adults, the asthma prevalence studies, published so far were done on military service conscripts[16], in primary care units [17,18], using postal questionnaires [19,20] or using postal questionnaire followed by a clinical visit [21]. A nationwide telephone survey conducted by the *Observatório Nacional de Saúde* (ONSA) [22] in 2004 only included adults and asthma was addressed only in a single question among many other regarding chronic diseases. The Portuguese National Health Survey of 2005/2006 [23] included participants from all age groups, but the focus of the study were chronic diseases in general and not asthma. Thus, none of the published studies on asthma could give us truly generalizable prevalence estimates for the Portuguese population.

In 2010, we conducted the first Portuguese National Asthma Survey - *Inquérito Nacional sobre Asma* (INAsma), in collaboration with *Sociedade Portuguesa de Alergologia e Imunologia Clínica* and *Sociedade Portuguesa de Pneumologia,* by appointment of the Portuguese Health Directorate. It comprised two phases. In the first phase, the aim was to estimate the prevalence of current asthma and in the second phase, the proportion of asthmatic patients with controlled disease.

In this article we describe the first phase of INAsma aiming to estimate the prevalence of current asthma in the Portuguese population and to assess the association between ‘Current asthma’ and comorbidities such as upper airways disease.

## Methods

### Study design

This prevalence study was a cross-sectional, population-based, nationwide telephone interview survey including all municipalities of Portugal.

The study was approved by a Hospital Ethics Committee (*Comissão de Ética do Hospital de São João, Porto)*. All participants gave oral informed consent and were informed that they could abandon the study whenever they pleased, without any implication for their healthcare. Data confidentiality was guaranteed by storing personal information separately from the study data.

We followed the STROBE statement guidelines for reporting observational studies [24].

### Setting

Portugal is a country situated on the Iberian Peninsula, in south-western Europe. It has seven regions including the islands and 308 municipalities (NUTS II). The estimated population in 2011 national census was 10 555 853 inhabitants. Most of the urban population is located in littoral regions of the country, which are more industrialized than interior regions.

### Sample size

The sample size calculation was based in confidence intervals for proportions. As so, for a prevalence of 6% [25] and for the narrow error margin of 0.015, a sample of 1067 individuals would be enough. However, the sample size of this study was calculated considering the two phases of the research project. In the first phase, we aimed to estimate current asthma prevalence in the Portuguese population. In the second phase we aimed to estimate the proportion of asthma patients that have their disease controlled. Thus, in phase 2 of the project, to estimate the proportion of controlled asthma patients with a margin of error of 3% and a 95% confidence level, assuming a proportion of 20% of uncontrolled asthma patients in the population, we needed a sample of at least 554 asthma patients identified in the first phase of the project. Assuming a loss of patients’ follow-up of 20%, we needed to recruit at least 665 asthma patients during the first phase. Within these premises, and assuming a prevalence of 6% in the Portuguese population [25] and a margin of error of 0.65% we needed a sample of at least 6000 persons from the general Portuguese population willing to participate in the first phase of the project.

### Participants

The target population was the Portuguese general population and the available population included all individuals living in Portugal, in households with a landline telephone (sampling frame). To obtain a representative sample of the population, a stratified cluster sampling design was used.

First, all municipalities were used as natural strata; in each municipality a sample of households with landline telephone numbers was selected with a probability proportional to municipality population as estimated in the 2001 National Census. The target number of households was set at 6 103. The sample of households was derived from the directory listed in residential *White Pages* from 2010. To draw a sample of telephone numbers in a municipality a list of all telephone numbers in that municipality was compiled. From the whole list of each municipality, a sample of household’s telephone numbers was randomly selected. Because part of the selected telephone numbers are from companies or are not allocated, 4 lists were randomly selected for each municipality, in order to allow for substitution of non-residential telephone numbers. A total of 24 412 telephone numbers were retrieved.

Next, and after the selection and identification of a residential number, one participant was randomly selected in each household. After identification of all the residents in the household, the selected participant in each household was the last person having his/her birthday. When the selected individual was younger than 15 years old the respondent was the usual caregiver. Individuals were excluded if they did not understand spoken Portuguese or had cognitive or physical conditions that could hamper the interview. In the final 20% of the sample, an oversampling strategy of males and younger age subjects was used to correct the common overrepresentation of participants from the female sex and older age groups observed on an interim analysis.

### Instruments and data collection

The main instrument for data collection was the Portuguese version of the 21-item questionnaire used in the GA^2^LEN survey. This questionnaire includes the ECHRS questions on asthma symptoms [6,26]. A few additional questions were added, mostly regarding socio-educational variables and use of healthcare resources.

A private company administered the questionnaire through Computer Assisted Telephone Interviews (CATI) performed by trained and experienced interviewers. Interviews were conducted between March and May of 2010, mostly between 17:00–22:00 h in weekdays and 11:00–22:00 h in weekends and holidays. Each telephone number was not abandoned before a minimum of ten attempts in different occasions. The interviews had a mean duration of 15 minutes.

To minimize other potential biases in data collection, several quality assurance measures were followed: interviewers were selected based on their previous experience on health-related data collection; each question was discussed in training sessions held between researchers and all interviewers; a research assistant was present in the setup, training and daily work of the interviewers, motivating and checking the compliance with the standardized operational procedures; data validity was periodically verified soon after being collected and custom statistic algorithms were used to detect extreme, illogical and missing values; the clarity of the questionnaire and its telephonic administration was assessed in a pilot study with 25 individuals before starting the data collection.

### Variables

The primary outcome, common co-morbidities and confounders were defined as follows:

*Current asthma*: positive answer to the question “Have you ever had asthma?” *and* at least one of 3 symptoms in the last 12 months: wheezing, waking with breathlessness or having an asthma attack.

*Lifetime asthma:* positive answer to the question “Have you ever had asthma?”

*Diagnosed asthma*: positive answer to the questions “Have you ever had asthma?” *and* “Are you taking any medication for asthma?”

*Rhinitis*: positive answer to the question “Do you have any nasal allergies, including hay fever?” Further classification of rhinitis in intermittent, persistent, mild and moderate/severe was done according to ARIA using GA^2^LEN survey questions [27].

*Sinusitis*: positive answer to questions “Have you been diagnosed as having chronic sinusitis by a doctor?” *and* “Have you felt sinus pressure, pain around the eyes or nose, for more than 12 weeks in last 12 months?”

*Chronic bronchitis:* positive answer to questions “Did you have phlegm when coughing for at least 3 months in the last year?” *and* smoked more than 10 Packs-year *and* be at least 40 years old.

*Eczema/atopic dermatitis:* Positive answer to “Have you ever had eczema or skin allergy?”

*Drug allergy:* Positive answer to “Have you been diagnosed as having drug allergy by a doctor?”

*Food allergy:* Positive answer to “Have you been diagnosed as having food allergy by a doctor?”

*Wheeze*: Positive answer to “Did you have wheezing or whistling in your chest in the last 12 months?”

*Asthma attack*: Positive answer to “Did you have an asthma attack in the last 12 months?”

*Smokers* reported smoking at least one cigarette every day for one year*; Ex-smokers* reported having quit smoking for more than one month; *Non-smokers* reported neither smoking nor ex-smoking. *Packs-year* is the number of cigarettes smoked per day / 20 * number of years smoking.

*Environmental Tobacco Smoke:* Positive answer to “Does anyone smokes inside your home?”

*Heart disease*: Positive answer to “Have you a heart condition?”

### Statistical analysis

The estimates from the sample were weighted so they could be generalized to the target population. A two-stage stratified sampling design was implemented using the complex sampling module of IBM SPSS Statistics version 19 (2010 SPSS, Inc. an IBM Company). First, a simple random sampling without replacement was used for selecting a random sample of households with landline telephone within each stratum (municipality). Second, within each selected household, one eligible household resident was randomly selected using simple random sampling without replacement. Two types of weights were used: first, weights were used to adjust for the sampling design taking into account the probability of selection of each subject and second, post-stratification weights were used to adjust for the true sex and age distribution of the target population (weights took into account sex and 5-year age strata in each sampling stratum), thus partially correcting for nonresponse and noncoverage bias.

Categorical variables were described with absolute frequencies, proportions and 95% Confidence Interval (95%CI). Comparisons of proportions were tested with Pearson Chi-Square for complex samples. A p-value of <0.05 was considered as statistically significant. Univariate analysis was used to assess associations between ‘Current asthma’ and rhinitis, sinusitis, chronic bronchitis and non-respiratory allergic disease. In order to have a more thorough understanding of the factors affecting its distribution and risk, analysis of factors associated with ‘Current asthma’ were performed using univariate and multivariate weighted logistic regression modelling. In the multivariate logistic regression models, the dependent variable was presence of ‘Current asthma’. Model goodness-of-fit was assessed by the Hosmer-Lemeshow test [28]. Discriminative/predictive power of the model was evaluated by ROC curve analysis. Results are presented as odds ratios (OR) for each category as compared with a predefined reference category and their respective 95% Confidence Intervals (95%CI). For the initial model all the covariates with p-value of <0.25 were included. The final multiple logistic regression model included the independent variables age, education level, Environmental Tobacco Smoke (ETS), rhinitis plus sinusitis, rhinitis without sinusitis, sinusitis without rhinitis, eczema, drug allergy and food allergy.

Statistical analyses were performed using IBM SPSS Statistics version 19 (2010 SPSS, Inc. an IBM Company).

## Results

### Participants

**Figure 1:**
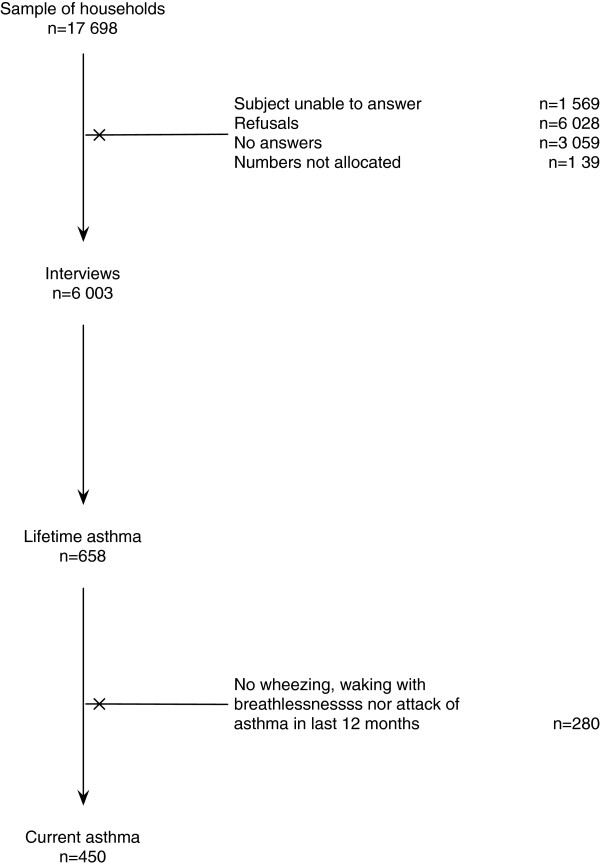
**Participants flowchart.** From the 17 698 households contacts, 6 003 participants were included in the study; 658 classified as having ‘Lifetime asthma’ and 450 as having ‘Current asthma’.

Of 17 698 contacts, 6 003 subjects completed the interview (Figure1). The sample response rate was 40%; the corrected response rate was 50%. The respondent was the usual caregiver in 76.7% of the participants younger than 15 years old. Participants’ characteristics are summarized in Table1. There were no missing data for the variables that comprised the definition of ‘Current asthma’. 

**Table 1:** Socio-demographic characteristics of the participants by ‘Current asthma’ status

		**Current asthma**	
	**Total (n = 6 003)**	**Yes (n = 450)**	**No (n = 5 553)**
**Municipality**			
Urban	4593 (76.5)	337 (74.9)	4256 (76.6)
Rural	1410 (23.5)	113 (25.1)	1297 (23.4)
**Female, n (%)**	3438 (57.3)	282 (62.7)	3156 (56.8)
**Age groups, n (%)**			
<18 years old	716 (11.9)	56 (12.4)	660 (11.9)
18-65 years old	3104 (51.7)	211 (46.9)	2893 (52.1)
>65 years old	2178 (36.3)	183 (40.7)	1995 (36.0)
**BMI**^**†**^**, n(%)**			
Underweight (<18)	253 ( 5.1)	18 ( 4.9)	235 ( 5.1)
Normal weight (18–25)	2179 (43.9)	140 (38.4)	2039 (44.3)
Overweight (25–30)	1825 (36.7)	128 (35.1)	1697 (36.9)
Obese (>30)	710 (14.3)	79 (21.6)	631 (13.7)
**Education level**^**‡**^**, n(%)**			
<9 years	3907 (65.5)	312 (69.6)	3595 (65.1)
9-12 years	1175 (19.7)	85 (19.0)	1090 (19.7)
>12 years	732 (12.3)	40 (8.9)	692 (12.5)
**SES**^**††**^**, n(%)**			
Low	1289 (22.0)	111 (25.2)	1169 (21.7)
Medium	4137 (71.1)	1169 (21.7)	3831 (71.2)
High	405 ( 7.0)	23 ( 5.2)	382 ( 7.1)
**Smoking status, n(%)**			
Non-smoker	4291 (71.5)	333 (74.0)	3958 (71.3)
Ex-smoker	971 (16.2)	71 (15.8)	900 (16.2)
Current smoker	741 (12.3)	46 (10.2)	695 (12.5)
**Unit Packs-year, n(%)**			
≤10 Packs-year	4799 (79.9)	368 (82.3)	4431 (80.5)
>10 Packs-year	1150 (19.2)	79 (17.7)	1071 (19.5)
**ETS**^**‡‡**^**, n(%)**			
No	4843 (80.7)	347 (77.1)	4496 (81.0)
Yes	1160 (19.3)	103 (22.9)	1057 (19.0)

†Body Mass Index. ^‡^A total of 153 (2.5%) children were pre-schoolers (not shown). ^††^Socioeconomic Status was categorized in high (A social class), medium (B and C social classes) and low (D social class) based on occupation and school education of the person who contributes more for the household income. ^‡‡^Environmental Tobacco Smoke.

### Prevalence of asthma

The prevalence of ‘Diagnosed asthma’ was 5.0% (95%CI 4.2-5.8) and the ‘Lifetime asthma’ prevalence was 10.5% (95%CI 9.5-11.6). In those with ‘Lifetime asthma’, 72.8% had their first asthma attack before 18 years old, 25.5% between 18 and 64 years old and 1.7% after 65 years old.

#### Current asthma

The prevalence of ‘Current asthma’ was 6.8% (95%CI 6.0-7.7). Using GA^2^LEN definition for asthma [7], our prevalence estimate was 7.8% (95%CI 7.0-8.8).

‘Current asthma’ prevalence was similar in men and women and in all age groups (Table2). The prevalence of asthma was similar in all body mass index (BMI) groups, with tendency to be higher in obese people: 7.7% (95%CI 2.2-23.3) in underweight group; 5.7% (95%CI 4.3-7.4) in normal weight; 6.4% (95%CI 5.1-8.1) in overweight and 8.7% (95%CI 6.5-11.4) in obese. There was no association between being overweight or obese and ‘Current asthma’ (Crude OR 1.13, 95%CI 0.78-1.64 and 1.54, 95%CI 1.01-2.34, respectively).

**Table 2:** Prevalence of ‘Current asthma’ and logistic regression models with crude and adjusted Odds Ratio (OR)

	**Current asthma % (95%CI)**	**Crude OR (95%CI)**	**Adjusted OR (95% CI)**^§^
**All Portuguese Population**	6.8 ( 6.0- 7.7)		
**NUTS II**			
North	6.7 ( 5.5- 8.2)	1.00 (Ref)	
Centre	6.2 ( 4.6- 8.3)	0.92 (0.63-1.35)	
Lisbon	6.8 ( 5.4- 8.6)	1.01 (0.72-1.42)	
Alentejo/Algarve	7.9 ( 5.4-11.3)	1.18 (0.75-1.87)	
Madeira/Azores	6.6 ( 3.9-10.9)	0.98 (0.55-1.77)	
**Municipality**			
Urban	6.5 ( 5.6- 7.4)	1.00 (Ref)	
Rural	7.7 ( 5.9-10.1)	1.22 (0.87-1.69)	
**Gender**			
Female	7.2 ( 6.0- 8.5)	1.00 (Ref)	
Male	6.3 ( 5.2- 7.6)	0.87 (0.66-1.14)	
**Age groups**			
<18 years old	7.2 ( 5.4- 9.5)	1.00 (Ref)	1.00 (Ref)
18-65 years old	6.3 ( 5.3- 7.5)	0.86 (0.61-1.23)	0.94 (0.63-1.40)
>65 years old	8.0 ( 6.7- 9.5)	1.11 (0.78-1.59)^*^	**1.51 (1.01-2.27)**^§*^
**Education level**^**†**^			
<9 years	7.1 ( 6.1- 8.2)	1.00 (Ref)	1.00 (Ref)
9-12 years	7.4 ( 5.6- 9.7)	1.05 (0.75-1.47)	0.92 (0.64-1.32)
>12 years	4.5 ( 3.0- 6.6)	0.62 (0.40-0.96)	**0.44 (0.28-0.70)**^§**^
**SES**^**‡**^			
Low	6.9 ( 5.3- 8.9)	1.00 (Ref)	
Medium	6.9 ( 5.9- 8.0)	1.00 (0.73-1.37)	
High	6.3 ( 3.8-10.3)	0.91 (0.50-1.66)	
**Smoking status**			
Non-smoker	6.9 ( 6.0- 8.0)	1.00 (Ref)	
Ex-smoker	6.3 ( 4.7- 8.5)	0.91 (0.63-1.30)	
Current smoker	6.3 ( 4.3- 9.1)	0.90 (0.59-1.34)	
**ETS**^**††**^			
No	6.1 ( 5.3- 7.0)	1.00 (Ref)	1.00 (Ref)
Yes	**8.6 ( 6.7-11.0)**	1.45 (1.06-1.97)^*^	**1.53 (1.09-2.16)**^§***^
**Chronic Bronchitis**^**‡‡**^			
No	6.5 (5.7-7.4)	1.00(Ref)	
Yes	14.6 (9.8-21.2)	2.23 (1.38-3.59)	
**Rhinitis plus Sinusitis**			
No	6.4 ( 5.6- 7.3)	1.00 (Ref)	1.00 (Ref)
Yes	**39.1 (24.9-55.4)**	9.34 (4.75-18.35)^**^	**13.93 (6.60-29.44)**^**§§**^
**Rhinitis without Sinusitis**			
No	4.6 ( 3.8- 5.5)	1.00 (Ref)	1.00 (Ref)
Yes	**14.9 (12.6-17.6)**	3.67 (2.79-4.82)^**^	**3.87(2.90-5.18)**^**§§**^
**Sinusitis without Rhinitis**			
No	6.7 ( 5.9- 7.6)	1.00 (Ref)	1.00 (Ref)
Yes	**8.7 ( 3.3-21.2)**	1.33 (0.47-3.76)^**^	2.01 (0.64-6.34)
**Eczema**			
No	5.1 ( 4.3- 6.1)	1.00 (Ref)	1.00 (Ref)
Yes	**11.5 ( 9.6-13.7)**	2.41 (1.84-3.17)^**^	**2.00 (1.50-2.66)**^**§§**^
**Food allergy**			
No	6.3 ( 5.5- 7.1)	1.00 (Ref)	1.00 (Ref)
Yes	**15.7 (10.8-22.3)**	2.79 (1.77-4.40)^**^	**1.89 (1.15-3.10)**^§§*^
**Drug allergy**			
No	6.3 ( 5.5- 7.3)	1.00 (Ref)	1.00 (Ref)
Yes	**11.9 ( 8.7-16.1)**	2.00 (1.38-2.92)^**^	1.30 (0.86-1.95)

^† ‘^Pre-schoolers’ and <9 years were merged. ^‡^Socioeconomic status was categorized in high (A social class), medium (B and C social classes) and low (D social class) based on occupation and school education of the person who contributes more for the household income. ^††^Environmental Tobacco Smoke. ^‡‡^Chronic bronchitis was defined as positive answer to questions “Did you have phlegm when coughing for at least 3 months in the last year?” and smoked more than 10 Packs-year and be at least 40 years old. *p < 0.25 **p < 0.001. § Final multiple logistic regression model included age, education level, ETS, , rhinitis plus sinusitis, rhinitis without sinusitis, sinusitis without rhinitis, eczema, drug allergy and food allergy as risk factors for Current asthma. §*p = 0.006 §**p = 0.002 §***p = 0.015 §§p < 0.001 §§*p = 0.012. Significant results in bold. OR – odds ratio.

Asthma was more frequent in people who did not smoke or smoked less than 10 packs-year (7.1%, 95%CI 6.2-8.2) than in those who smoked more than 10 packs-year (5.2%, 95%CI 3.9-6.8; p = 0.042). Those who smoke more have lower risk of asthma (Crude OR 0.71, 95%CI 0.51-0.99). This tendency was significant after adjustments of unit packs-years for gender, age, BMI, education level and for ETS (data not shown).

In the multiple logistic regression, the risk of ‘Current asthma’ was assessed for age, education level, ETS, rhinitis with sinusitis, rhinitis without sinusitis, sinusitis without rhinitis, eczema, drug allergy and food allergy (Adjusted OR, Table2). The Hosmer-Lemeshow statistic revealed good fitting (p = 0.259) and the model’s predictive power was acceptable (AUC = 0.74, 95%CI 0.71-0.76).

### Comorbidities

Of the subjects with ‘Current asthma’, 5.7% (95%CI 3.8-8.5) also have chronic bronchitis (Table3). More than half of the subjects with ‘Current asthma’ also had rhinitis (52.3%, 95%CI 45.8-58.6), of which most were classified as intermittent moderate/severe (29.9%, 95%CI 24.4-36.0) (Table3). Rhinitis and Sinusitis alone had a strong association with asthma (Crude OR 3.67, 95%CI 2.79-4.82 and 1.33, 95%CI 0.47-3.76, respectively). Association between upper airway diseases and asthma was stronger in patients with both rhinitis and sinusitis (Crude OR 9.34, 95%CI 4.75-18.35 and Adjusted OR 13.93, 95%CI 6.60-29.44, Table2).

**Table 3:** Asthma comorbities for ‘Current asthma’ subjects (n = 450) and for the total sample (n = 6003)

	**Current asthma subjects, % (95%CI)**	**Total sample, % (95%CI)**
**Upper airways disease**		
None (Rhinitis-/Sinusitis-)	46.9 (40.5-53.4)*	77.2 (75.7-78.7)
Rhinitis	52.3 (45.8-58.6)*	22.1 (20.7-23.6)
Mild	14.0 (10.4-18.8)*	8.3 ( 7.4- 9.3)
Intermittent	12.1 ( 8.6-16.7)	7.4 ( 6.6- 8.4)
Persistent	1.9 ( 1.0- 3.8)	0.9 ( 0.6- 1.2)
Moderate/Severe	38.2 (32.3-44.5)*	13.8 (12.6-15.1)
Intermittent	29.9 (24.4-36.0)	11.1 (10.1-12.3)
Persistent	8.3 ( 5.7-12.0)	2.7 ( 2.2- 3.3)
Rhinitis+ /Sinusitis-	46.7 (40.4-53.2)*	21.2 (19.8-22.6)
Sinusitis	6.4 ( 4.3- 9.4)*	1.6 ( 1.2- 2.1)
Rhinitis - /Sinusitis+	0.9 ( 0.3- 2.2)	0.7 ( 0.4- 1.0)
Both (Rhinitis+/Sinusitis+)	5.5 ( 3.5- 8.5)*	1.0 ( 0.7- 1.3)
**Chronic bronchitis**	5.7 ( 3.8- 8.5)*	2.7 ( 2.2- 3.2)
**Non-respiratory allergic disease**	55.4 (48.9-61.7)*	33.0 (31.4-34.7)
Eczema/atopic dermatitis	44.3 (38.1-50.8)*	26.1 (24.6-27.7)
Drug allergy	13.3 ( 9.8-17.9)*	7.5 ( 6.7- 8.5)
Food allergy	12.2 ( 8.3-17.6)*	5.3 ( 4.5- 6.1)

(−) indicates absence; (+) indicates presence; *p < 0.001.

### Subgroup analysis

**Figure 2:**
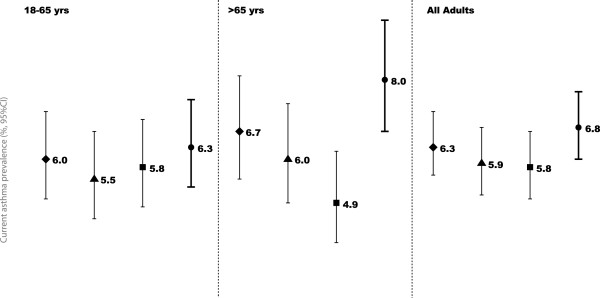
**Current asthma prevalence in subgroups of adults by symptoms possible to affect the estimates.** Symbols represent the prevalence of ‘Current asthma’ in people: ♦ without chronic bronchitis; ▲who smoked ≤10 packs-year; ■ without self reported heart disease and ● all participants of the age group.

To exclude possible confounding of asthma related symptoms with symptoms from self-reported heart disease, heavy smoking habits (smoking more than 10 packs-year) and chronic bronchitis a subgroups analysis was performed. Chronic bronchitis was the condition to have less impact on prevalence of ‘Current asthma’, whereas self-reported heart disease was the condition with most impact (Figure2). However, only in the older adults subgroup the participants without self-reported heart disease had a significantly lower prevalence of current asthma (older adults without heart disease 4.9%, 95%CI 3.9-6.2; all older adults 8.0%, 95%CI 6.7-9.5).

### Prevalence of symptoms

The most common respiratory symptom in the group with ‘Current asthma’ was wheeze (89%, 95%CI 85.8-91.6), followed by nocturnal symptoms (75.8%, 95%CI 70.8-80.2) (Table4). Of those who reported ‘Wheeze’ and ‘Wheeze with breathlessness’ (Positive answer to wheeze and “Did you have breathlessness when the wheezing sound present?”) and ‘Wheeze without a cold’ (Positive answer to wheeze and “Did you have wheezing without a could?”), 42.2% (95%CI 36.7-47.8) denied having asthma.

**Table 4:** Prevalence of self-reported symptoms in last 12 months for ‘Current asthma’ subjects(n = 450) and total sample(n = 6003)

	**Current asthma subjects, % (95%CI)**	**Total sample, % (95%CI)**
**Asthma Symptoms**		
Wheeze	89.0 (85.8-91.6)*	18.1 (16.8-19.4)
Nocturnal symptoms	75.8 (70.8-80.2)*	35.6 (33.9-37.3)
Waking with breathlessness	36.4 (30.7-42.6)*	6.8 ( 5.9- 7.7)
Waking with tightness in the chest	39.2 (33.3-45.4)*	11.9 (10.9-13.1)
Waking with cough	55.7 (49.1-62.0)*	28.0 (26.4-29.7)
Attack of asthma	46.9 (41.4-52.4)*	3.2 ( 2.6- 3.9)
**Sputum for at least 3 months**	45.4 (39.0-51.9)*	18.5 (17.1-19.9)
**Blocked nose**	27.5 (22.4-33.3)*	12.5 (11.4-13.7)
**Sinus pressure**	25.0 (20.4-30.3)*	11.2 (10.2-12.4)

*p < 0.001.

### Healthcare resources

About 243 000 (2.4%, 95%CI 1.9-2.9) Portuguese were hospitalized at least once in their lifetime because of asthma. In Portuguese subjects with ‘Current asthma’, 25.6% were hospitalized because of their asthma (Table5). Current use of asthma medication (inhaled, nebulized and/or oral medication) was reported by 60.1% of Portuguese subjects with ‘Current asthma’, 35.2% used inhaled controller medications and 21.6% takes reliever medications only (Table5).

**Table 5:** Healthcare resources (Medication, diagnosis and hospitalization) for ‘Current asthma’ subjects(n = 450) and for total sample(n = 6003)

	**Current asthma subjects, % (95%CI)**	**Total sample, % (95%CI)**
**Hospitalization because of asthma**	25.6 (20.4-31.5)	2.4 ( 1.9- 2.9)
**Lung function tests**	57.4 (50.9-63.7)	23.6 (22.2-25.2)
**Allergy tests**		
Any allergic test	65.7 (59.5-71.5)	25.2 (23.7-26.8)
Skin-prick test	55.2 (48.7-61.4)	17.9 (16.6-19.3)
Blood test	46.7 (40.3-53.1)	16.0 (14.7-17.4)
**Asthma medication**		
Any asthma medication	60.1 (53.5-66.2)	5.0 ( 4.2- 5.8)
Inhaled Controller	35.2 (29.2-41.6)	2.9 ( 2.4- 3.6)
Only inhaled Reliever	21.6 (16.7-27.5)	1.5 ( 1.1- 1.9)
Nebulized aerosols	36.3 (30.2-42.9)	2.5 ( 2.0- 3.1)
Nebulized aerosols without controller^†^	17.6 (13.2-23.0)	1.2 ( 0.9- 1.6)
**Antihistamine medication**		
Nasal spray	24.9 (19.8-30.7)	1.7 ( 1.3- 2.1)
Oral medication	45.3 (39.1-51.8)	3.1 ( 2.6- 3.7)

^†^Includes the participants using nebulized aerosols but not inhaled controller medication.

## Discussion

In the present study the estimated prevalence of ‘Current asthma’ in the Portuguese population was 6.8%. This study also showed a strong association of asthma with rhinitis and sinusitis, in agreement with the known risk of the chronic disease of upper airways for asthma [27].

A limitation of the method used is the increasing preference for mobile phones over residential telephones. However, the use of mobile phone does not allow stratification by geographical region; reduces control over the sampling method and has a lower response rate [29]. This study has the intrinsic limitations of a telephone survey on asthma; such as the impossibility to determine causality factors without risking biased inferences. Other limitations are is the fact that the only data available by the time of the data collection was the National Census of 2001; demographic changes in the years in between could influence the sampling size; and the relatively low response rate, still was among the highest obtained in Portuguese nationwide telephone surveys [30,31]. In other countries the asthma prevalence and outcomes are different between ethnicities. It would be interesting to compare the prevalence of asthma in the people with different ethnic backgrounds in the Portuguese population. However, in Portugal in what concerns ethnicity, serious limitations on data collection are imposed and no official data are available.

Bias and confounding are limitations of all epidemiological studies. To address this, we took into account possible information and selection bias and adjusted for conditions with similar symptoms. Chronic Obstructive Pulmonary Disease (COPD) can be difficult to differentiate from asthma in surveys [32], especially when the proportion of elders with respiratory symptoms is high and is associated with asthma, as we have observed. Nevertheless, we believe misclassification of COPD patients had a limited effect on our prevalence estimates. The analysis of the participants without ‘Chronic bronchitis’ (phlegm when coughing for at least 3 months in the last year and smoked more than 10 packs-year and is at least 40 years old) didn’t change asthma prevalence estimates. After these adjustments, the prevalence of asthma was lower in elders without self-reported ‘heart disease’. This overestimation of asthma symptoms in people with heart disease may indicate a possible bias or confounding of the two conditions, since heart disease is another cause of recurrent respiratory symptoms [1]. In spite of these limitations, this is the only Portuguese study using a study-proved questionnaire on asthma that includes a large sample size from all municipalities and all age groups and which estimates represent the prevalence in Portuguese population.

An additional clinical evaluation including lung function tests would improve the accuracy of asthma classification [3,4]. We designed and intend to proceed with a clinical evaluation in a subsample of the participants that will allow us to assess the effect of the survey misclassification on asthma prevalence estimates.

### Current asthma

GA^2^LEN definition of asthma included the symptoms ‘wheezing’; ‘waking with a feeling of tightness in the chest’; ‘waking with breathlessness’ and ‘waking with cough’. Using this definition our estimate was 7.8% (95%CI 7.0-8.8). However, our definition of ‘Current asthma’ uses a more conservative combination of questions from the GA^2^LEN survey. The selection of the questions aimed to improve the specificity, namely by including ‘asthma attack’ and ‘waking with breathlessness’ [33-35] (the less frequent nocturnal asthma-related symptom) and not including ‘waking with a feeling of tightness in the chest’ or ‘waking with cough’. Our prevalence estimate using this definition of ‘Current asthma’ (6.8%, 95%CI 6.0-7.7), was comparable to those found in GA^2^LEN survey for Sweden (Gothenburg, 7.1%), Poland (Krakow 7.1% and Lodz 6.0%), Netherlands (6.4%), Belgium (7.6%) and Germany (Brandenburg 6.3%) but inferior to Coimbra estimates (16.8%) [7].

A direct comparison of our prevalence estimates with other studies previously done in Portugal is not straightforward given the differences in the methods and operational definitions used. Most of the asthma studies in Portugal were done on school-attending children and teenagers from selected cities or regions, in these the prevalence of current asthma ranged from 3% to 8%[8,10,13,19,21].

The ISAAC study in Portugal assessed the prevalence of asthma in four cities. For children aged 6–7 years old the global prevalence of asthma in last 12 months was 13% (in Lisboa, Portimão and Funchal); for teenagers with 13–14 years old the global prevalence of current asthma ranged between 9% in 1995 and 12% in 2002 (in Lisboa, Porto, Portimão, Funchal)[5].

The ECRHS I included data were collected in two Portuguese cities (Porto and Coimbra) [6]. A total of 3850 participants responded to the postal questionnaire, and the estimated prevalence of asthma diagnosis was 4% in Porto and 6% in Coimbra [6].

None of the previously published studies exclusively on asthma conducted in Portugal can be confidently generalized to the Portuguese population because of methodological reasons (sampling methods, data collection or asthma definition used).

The only study done in the entire population addressing both children and adults was the Portuguese National Health Survey of 2005/2006. In this survey 3.5% (368 184) reported having had asthma in last 12 months [23], this is numerically inferior to the number of Portuguese ‘Current asthma’ estimated in the present study (695 000). This could suggest an increase of asthma prevalence commonly to other westernized countries [36-39]. However, the comparison of the two studies may be compromised due to the methodological differences stated above.

#### Participant characteristics and asthma

Asthma prevalence was similar in both genders and in all Portuguese regions, as observed in ONSA survey [22]. The higher prevalence of asthma in older adult population was also observed the Portuguese National Health Survey [23] and ONSA [22].

Obesity has been associated with asthma [1,40,41]. Using the Portuguese National Health Survey data, Moreira et al. couldn’t show that higher BMI is an increased risk for asthma [42]. Our results confirm this lack of association.

Additionally, lower educational level and SES has been linked to an increased risk of asthma [1,43,44]. A study in Portuguese adults, showed that the risk of asthma is associated with lower monthly income and tended to be higher in people with lower educational level [25]. In the present study, educational level was associated with asthma only after adjustment for the other variables. SES was not associated with asthma.

‘Current asthma’ prevalence tended to be higher in non-smokers or in those who smoked up to 10 packs-year, suggesting either underreporting of asthma symptoms by participants with tobacco-related diseases or that individuals with respiratory symptoms are less likely to become smokers or tend to quit smoking. Although this association is controversial, similar result was observed in a case–control study based on ECHRS survey data: smokers showed a 42% lower risk of asthma than did non-smokers (OR 0.58; 95% CI: 0.36 to 0.92) [45].

ETS may have an effect on asthma risk and on asthma patients [1]. In the present study, exposure to ETS at home is a significant risk factor for ‘Current asthma’.

#### Comorbidities

The association between asthma and rhinitis without sinusitis found in the present study (Crude OR 3.67, 95%CI 2.79-4.82) is similar to other European studies [46] supporting the concept of a frequent co-existence of asthma and rhinitis in the same patient [1,27]. In fact, rhinitis has been considered an important risk factor for asthma development and severity [46]. An association with sinusitis without rhinitis was also found (Crude OR 1.33, 95%CI 0.47-3.76), in agreement with the findings of a recent European study [7]. This association was stronger when both rhinitis and sinusitis were present, comparably to results from previous studies [7,47].

### Healthcare resources

Almost half of the people with ‘Current asthma’ are not receiving any asthma treatment, and a fifth is using only rescue medication. Furthermore, about 40% of people with wheeze denied having asthma. These facts seem to suggest the need for improvement of diagnosis and treatment of asthma as stated by the Global Alliance against Chronic Respiratory Diseases [48].

## Conclusion

In conclusion, 695,000 Portuguese have Current asthma, with a prevalence of 6.8%, and more than one million (10.5%) had Lifetime asthma. People who reported sinusitis and rhinitis had the strongest risk of having Current asthma.

## Abbreviations

BMI: Body mass index; CATI: Computer assisted telephone interviews; CI: Confidence interval; COPD: Chronic obstructive pulmonary disease; ECRHS: European community respiratory health survey; ETS: Environmental tobacco smoke; GA^2^LEN: Global allergy and asthma european network; INAsma: Inquérito nacional sobre asma; ISAAC: International study of asthma and allergies in childhood; ONSA: Observatório nacional de saúde; OR: Odds ratio; ROC: Receiver operating curve; SES: Socioeconomic status.

## Competing interests

The author(s) declare that they have no competing interests.

## Authors’ contributions

ASS participated in data collection, analysis and wrote the manuscript draft, LFA participated in study design, data analysis and reviewed the manuscript, RC participated in data collection, TJ, MMA and ABA participated in the study conception and reviewed the manuscript, ATB and CL provided parts of the questionnaire for data collection and reviewed the manuscript and JB provided critical review during the project and reviewed the manuscript, JAF is responsible for the INAsma project and participated in all stages and tasks. All authors have read and approved the final manuscript.

## References

[B1] Global Initiative for Asthma Global strategy for asthma management and prevention (update 2009) 2009

[B2] Bousquet J Khaltaev N Cruz AA Global surveillance, prevention and control of Chronic Respiratory Diseases: A comprehensive approach 2007 World Health Organization, Switzerland

[B3] Burney PG Luczynska C Chinn S Jarvis D The European Community Respiratory Health Survey Eur Respir J 1994 7 954 960 10.1183/09031936.94.07050954 8050554

[B4] Asher MI Keil U Anderson HR Beasley R Crane J Martinez F Mitchell EA Pearce N Sibbald B Stewart AW International Study of Asthma and Allergies in Childhood (ISAAC): rationale and methods Eur Respir J 1995 8 483 491 10.1183/09031936.95.08030483 7789502

[B5] Rosado Pinto J ISAAC - 20 anos em Portugal Acta Pediatr Port 2011 42 S35 S40

[B6] Burney P Chinn C Luczynska C Jarvis D Vermeire P Bousquet J Nowak D Prichard J de Marco R Rijcken B Variations in the prevalence of respiratory symptoms, selfreported asthma attacks, and use of asthma medication in the European Community Respiratory Health Survey (ECRHS) Eur Respir J 1996 9 687 6958726932

[B7] Jarvis D Newson R Lotvall J Hastan D Tomassen P Keil T Gjomarkaj M Forsberg B Gunnbjornsdottir M Minov J Asthma in adults and its association with chronic rhinosinusitis: The GA2LEN survey in Europe Allergy 2012 67 91 98 10.1111/j.1398-9995.2011.02709.x 22050239

[B8] Santos JM Aspectos epidemiológicos da asma pediátrica numa comunidade portuguesa 1993 Lisboa

[B9] Prata C Marto J Mouzinho I Menezes M Susano R Epidemiologic study of bronchial asthma in schoolchildren from the Azores (Faial) Acta Med Port 1994 7 541 544 7856460

[B10] Vicente O Rodrigues T Silva AM Tzer TS Barros H Prevalência de asma em estudantes das escolas secundárias portuguesas Arq Med 1995 9 90 92

[B11] Morais-Almeida M Câmara R Ornelas P Prevalência de asma brônquica e de atopia em crianças da Ilha da Madeira Rev Epidemiol 1996 2 39 40

[B12] Leiria Pinto P Asma brônquica e o adolescente. Conhecimentos e atitudes 1998 Universidade Nova de Lisboa, Faculdade Ciências Médicas, Lisboa

[B13] Barros H Pereira C Mateus P Asma em crianças dos 6 aos 9 anos. Um estudo populacional em duas cidades portuguesas (Porto e Viseu) Rev Port Imunoalergologia 1999 7 9 18

[B14] Falcão H Ramos E Marques JA Barros H Prevalence of asthma and rhinitis in 13 year old adolescents in Porto, Portugal Rev Port Pneumol 2008 14 747 768 19023493

[B15] Pegas PN Alves CA Scotto MG Evtyugina MG Pio CA Freitas MC Risk factors and prevalence of asthma and rhinitis among primary school children in Lisbon Rev Port Pneumol 2011 17 109 116 10.1016/j.rppneu.2011.01.004 21549669

[B16] Chieira C Loureiro AC Rodrigues VL Estudos epidemiológiocs alergológiocs numa população de mancebos (20 anos) Via Pneumológica 1990 1 67

[B17] Nunes C Ladeira S Albuquerque J Epidemiological study of asthma in schoolchildren J Med 1987 CXXM 2200 40 51

[B18] Correia-de-Sousa J Espirito-Santo M Colaco T Almada-Lobo F Yaphe J Asthma in an Urban Population in Portugal: A prevalence study BMC Publ Health 2011 11 347 10.1186/1471-2458-11-347 PMC3121634 21595928

[B19] Loureiro AC Chieira C Pereira AC Todo Bom A Faria E Alendouro P Tavares B Rodrigues VL Cardoso SM Robalo Cordeiro AJA Estudos Epidemiológicos da Asma Brônquica numa População Adulta Rev Port Imunoalergologia 1996 4 35 54

[B20] Marques JA Epidemiology of asthma in Portugal Arq Med 1993 7 116 120

[B21] Alves J Hespanhol V Magalhaes A Almeida J Marques JA Prevalence of asthma in the city of Porto Acta Med Port 1994 7 21 24 8184717

[B22] Branco MJ Nogueira P Contreiras T Report on prevalence estimates of some chronic diseases in mainland Portugal 2005 Observatório Nacional de Saúde, Lisboa

[B23] Instituto Nacional de Estatística/Inquérito Nacional de Saúde Inquérito Nacional de Saúde 2005/2006 2009 Lisboa

[B24] von Elm E Altman DG Egger M Pocock SJ Gotzsche PC Vandenbroucke JP The Strengthening the Reporting of Observational Studies in Epidemiology (STROBE) statement: guidelines for reporting observational studies J Clin Epidemiol 2008 61 344 349 10.1016/j.jclinepi.2007.11.008 18313558

[B25] Moreira P Moreira A Padrão P Delgado L The role of economic and educational factors in asthma: Evidence from the Portuguese Health Survey Public Health 2008 122 434 439 10.1016/j.puhe.2007.07.014 17923141

[B26] Bousquet J Burney PG Zuberbier T Cauwenberge PV Akdis CA Bindslev-Jensen C Bonini S Fokkens WJ Kauffmann F Kowalski ML GA2LEN (Global Allergy and Asthma European Network) addresses the allergy and asthma 'epidemic' Allergy 2009 64 969 977 10.1111/j.1398-9995.2009.02059.x 19392994

[B27] Bousquet J Khaltaev N Cruz AA Denburg J Fokkens WJ Togias A Zuberbier T Baena-Cagnani CE Canonica GW van Weel C Allergic Rhinitis and its Impact on Asthma (ARIA) 2008 update (in collaboration with the World Health Organization, GA(2)LEN and AllerGen) Allergy 2008 63 Suppl 86 8 160 18331513 10.1111/j.1398-9995.2007.01620.x

[B28] Hosmer D Lemeshow S Applied Logistic Regression 1989 New York

[B29] Brick JM Brick PD Dipko S Presser S Tucker C Yuan Y Cell phone survey feasibility in the us: Sampling and calling cell numbers versus landline numbers Public Opin Q 2007 71 23 10.1093/poq/nfl040

[B30] Correia S Dinis P Rolo F Lunet N Sampling procedures and sample representativeness in a national telephone survey: a Portuguese example Int J Public Health 2010 55 261 269 10.1007/s00038-009-0102-2 20013144

[B31] Correia S Dinis P Rolo F Lunet N Prevalence, treatment and known risk factors of urinary incontinence and overactive bladder in the non-institutionalized Portuguese population Int Urogynecol J Pelvic Floor Dysfunct 2009 20 1481 1489 10.1007/s00192-009-0975-x 19684999

[B32] Bateman ED Hurd SS Barnes PJ Bousquet J Drazen JM FitzGerald M Gibson P Ohta K O'Byrne P Pedersen SE Global strategy for asthma management and prevention: GINA executive summary Eur Respir J 2008 31 143 178 10.1183/09031936.00138707 18166595

[B33] Burney PG Laitinen LA Perdrizet S Huckauf H Tattersfield AE Chinn S Poisson N Heeren A Britton JR Jones T Validity and repeatability of the IUATLD (1984) Bronchial Symptoms Questionnaire: an international comparison Eur Respir J 1989 2 940 945 2606194

[B34] de Marco R Cerveri I Bugiani M Ferrari M Verlato G An undetected burden of asthma in Italy: the relationship between clinical and epidemiological diagnosis of asthma Eur Respir J 1998 11 599 605 9596109

[B35] Toren K Brisman J Jarvholm B Asthma and asthma-like symptoms in adults assessed by questionnaires. A literature review Chest 1993 104 600 608 10.1378/chest.104.2.600 7802735

[B36] Anderson HR Ruggles R Strachan DP Austin JB Burr M Jeffs D Standring P Steriu A Goulding R Trends in prevalence of symptoms of asthma, hay fever, and eczema in 12–14 year olds in the British Isles, 1995–2002: questionnaire survey BMJ 2004 328 1052 1053 10.1136/bmj.38057.583727.47 15028634 PMC403847

[B37] Toelle BG Ng K Belousova E Salome CM Peat JK Marks GB Prevalence of asthma and allergy in schoolchildren in Belmont, Australia: three cross sectional surveys over 20 years BMJ 2004 328 386 387 10.1136/bmj.328.7436.386 14962876 PMC341389

[B38] Pallasaho P Lundback B Laspa SL Jonsson E Kotaniemi J Sovijarvi AR Laitinen LA Increasing prevalence of asthma but not of chronic bronchitis in Finland? Report from the FinEsS-Helsinki Study Respir Med 1999 93 798 809 10.1016/S0954-6111(99)90265-2 10603629

[B39] Soriano JB Kiri VA Maier WC Strachan D Increasing prevalence of asthma in UK primary care during the 1990s Int J Tuberc Lung Dis 2003 7 415 421 12757040

[B40] van Huisstede A Braunstahl GJ Obesity and asthma: co-morbidity or causal relationship? Monaldi Arch Chest Dis 2010 73 116 123 21214041 10.4081/monaldi.2010.295

[B41] Beuther DA Weiss ST Sutherland ER Obesity and asthma Am J Respir Crit Care Med 2006 174 112 119 10.1164/rccm.200602-231PP 16627866 PMC2662903

[B42] Moreira P Moreira A Padrao P Delgado L Obesity and asthma in the Portuguese National Health Survey Allergy 2006 61 1488 1489 10.1111/j.1398-9995.2006.01182.x 17073885

[B43] Litonjua AA Carey VJ Weiss ST Gold DR Race, socioeconomic factors, and area of residence are associated with asthma prevalence Pediatr Pulmonol 1999 28 394 401 10.1002/(SICI)1099-0496(199912)28:6<394::AID-PPUL2>3.0.CO;2-6 10587412

[B44] Basagana X Sunyer J Kogevinas M Zock JP Duran-Tauleria E Jarvis D Burney P Anto JM Socioeconomic status and asthma prevalence in young adults: the European Community Respiratory Health Survey Am J Epidemiol 2004 160 178 188 10.1093/aje/kwh186 15234940

[B45] de Marco R Locatelli F Sunyer J Burney P Differences in incidence of reported asthma related to age in men and women. A retrospective analysis of the data of the European Respiratory Health Survey Am J Respir Crit Care Med 2000 162 68 74 10903222 10.1164/ajrccm.162.1.9907008

[B46] Cruz AA Popov T Pawankar R Annesi-Maesano I Fokkens W Kemp J Ohta K Price D Bousquet J Common characteristics of upper and lower airways in rhinitis and asthma: ARIA update, in collaboration with GA(2)LEN Allergy 2007 62 Suppl 84 1 41 17924930 10.1111/j.1398-9995.2007.01551.x

[B47] Guerra S Sherrill DL Martinez FD Barbee RA Rhinitis as an independent risk factor for adult-onset asthma J Allergy Clin Immunol 2002 109 419 425 10.1067/mai.2002.121701 11897985

[B48] Bousquet J Dahl R Khaltaev N Global alliance against chronic respiratory diseases Allergy 2007 62 216 223 10.1111/j.1398-9995.2007.01307.x 17298337

